# ERGA-BGE Genome of *Ailoscolex lacteospumosus* Bouché, 1969 – the enigmatic milky worm endemic to the Pyrenees

**DOI:** 10.12688/openreseurope.22136.1

**Published:** 2026-01-26

**Authors:** Marta Novo, Daniel Fernández Marchán, Sylvain Gérard, Alejandro Martínez Navarro, Rita Monteiro, Astrid Böhne, Thomas Marcussen, Torsten H Struck, Rebekah A Oomen, Laura Aguilera, Marta Gut, Francisco Câmara Ferreira, Jèssica Gómez-Garrido, Fernando Cruz, Tyler Alioto, Anna Lazar, Leanne Haggerty, Fergal Martin, Tom Brown

**Affiliations:** 1 Facultad de Ciencias Biológicas, Universidad Complutense De Madrid, Madrid, Spain; 2 Organismal and Evolutionary Biology, Faculty of Biological and Environmental Sciences, University of Helsinki, Helsinki, Finland; 3 Leibniz Institute for the Analysis of Biodiversity Change - Museum Koenig Bonn, Adenauerallee 127, Bonn, 53113, Germany; 4 Natural History Museum, University of Oslo, P.O. Box 1172, Blindern, Oslo, 0318, Norway; 5 Centre for Ecological & Evolutionary Synthesis, University of Oslo, Blindernveien 31, Oslo, 0371, Norway; 6 Department of Biological Sciences, University of New Brunswick Saint John, 100 Tucker Park Road, Saint John, E2K5E2, Canada; 7 Tjärnö Marine Laboratory, University of Gothenburg, Hättebäcksvägen 7, Gothenburg, 45296, Sweden; 8 Centre for Coastal Research, University of Agder, Universitetsveien 25, Kristiansand, 4630, Norway; 9 Centro Nacional de Análisis Genómico (CNAG), Baldiri Reixac 4, Barcelona, 08028, Spain; 10 Universitat de Barcelona, Barcelona, Spain; 11 European Molecular Biology Laboratory, European Bioinformatics Institute, Wellcome Genome Campus, Hinxton, Cambridge, UK; 12 Leibniz Institute for Zoo and Wildlife Research, Alfred-Kowalke-Straße 17, Berlin, 10315, Germany; 13 Berlin Center for Genomics in Biodiversity Research (BeGenDiv), Koenigin-Luise-Str 6-8, Berlin, 14195, Germany

**Keywords:** Ailoscolex lacteospumosus, genome assembly, European Reference Genome Atlas, Biodiversity Genomics Europe, Earth Biogenome Project, milky worm

## Abstract

*Ailoscolex lacteospumosus* represents one of the basal taxa of the earthworm family Hormogastridae and was initially placed in a different family, Ailoscolecidae, including just this species. Its genome can help unravel the evolution of this earthworm family, which is endemic to the Mediterranean and adapted to soils exposed to adverse conditions such as prolonged droughts. This species presents a very restricted distribution, endemic to the Pyrenees, and the genome will help study its conservation status as well as provide insights into earthworm evolution. A total of 17 contiguous chromosomal pseudomolecules were assembled from the genome sequence. This chromosome-level assembly encompasses 493 Mb, composed of 46 contigs and 24 scaffolds, with contig and scaffold N50 values of 15.1 Mb and 29.9 Mb, respectively.

## Introduction

**Figure 1.**
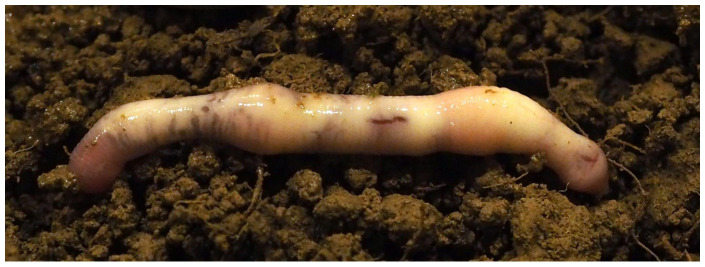
Example image of *Ailoscolex lacteospumosus*. Image taken by Sylvain Gérard.

*Ailoscolex lacteospumosus* Figure 1 & Bouché, 1969, is an earthworm within a monospecific genus and presents a very restricted distribution area, known only from a few localities covering a range of around 24 km in the region of Ariège, southern France ( Marchán *et al.*, 2018a). Initially placed in the family Ailoscolecidae ( Bouché, 1969), it has recently been included in the family Hormogastridae ( Marchán *et al.*, 2018b), following confirmation by phylogenomic analyses ( Novo *et al.*, 2016). It is a family endemic to the western Mediterranean, adapted to soils prone to drought and with the capacity of aestivation in summer ( Díaz Cosín *et al.*, 2006). Like other hormogastrids, it lacks dorsal pores and Morren glands and presents closely paired chaetae and an anterior position of the clitellum. *Ailoscolex* has two anterior gizzards and a multilamellar typhlosole (three lamellae). The male pore is displaced to a more posterior position in segment 22 and presents a shorter clitellum when compared to most hormogastrids (9-11 segments). It has two pairs of globular spermathecae ( Bouché, 1969).

*Ailoscolex* is one of the basal taxa of Hormogastridae ( Novo *et al*., 2016) and therefore, its genome can provide valuable insights into the evolution of these Mediterranean worms. Furthermore, its extremely reduced distribution range and unique morphology highlight the importance of assessing its conservation status and implementing protection measures.

The conservation status of *Ailoscolex lacteospumosus* has not been formally assessed and therefore it is not listed in the IUCN Red List or any other red list. However, it presents a very limited distribution, which calls for the need to conserve this species.

*Ailoscolex lacteospumosus* is an endogeic earthworm species, which means that it lives in the deepest layers of the soil and builds horizontal galleries. These galleries improve water infiltration and aeration and through its feeding and burrowing, it has a significant impact on nutrient cycling, organic matter decomposition and soil structure. It therefore plays a fundamental role in terrestrial ecosystems, as described for earthworms ( Lavelle *et al*., 2006).

The generation of this reference resource was coordinated by the European Reference Genome Atlas (ERGA) initiative’s Biodiversity Genomics Europe (BGE) project, supporting ERGA’s aims of promoting transnational cooperation to promote advances in the application of genomics technologies to protect and restore biodiversity ( Mazzoni *et al*., 2023).

## Materials & methods

ERGA's sequencing strategy includes Oxford Nanopore Technology (ONT) and/or Pacific Biosciences (PacBio) for long-read sequencing, along with Hi-C sequencing for chromosomal architecture, Illumina Paired-End (PE) for polishing (i.e. recommended for ONT-only assemblies), and RNA sequencing for transcriptomic profiling, to facilitate genome assembly and annotation.

### Sample and sampling information

On 7th November 2023, 12 adult, hermaphroditic samples of *Ailoscolex lacteospumosus* were sampled by Daniel Fernández Marchán, Sylvain Gérard, and Alejandro Martínez Navarro. The species identification was confirmed via morphology and barcoding. The specimen was sampled by digging by hand in a pasture in Montesquieu-Avantès, Ariège, France. The specimen's tissues (head, anterior body and posterior body) were dissected by placing them on a glass petri dish over dry ice, kept immediately in the barcoded tubes and placed in liquid nitrogen until storage at -80. Permits are not required for sampling and sequencing of this worm, as indicated by the French Ministère de la Transition écologique, de la Biodiversité, de la Forêt, de la Mer et de la Pêche.

### Vouchering information

Physical reference materials for the here sequenced specimen have been deposited in MNCN ( https://www.mncn.csic.es/es), under the accession number MNCN16.1/19304.

Frozen reference tissue material of bodywall is available from the same individual at the MNCN ( https://www.mncn.csic.es/es) under the voucher ID MNCN-ADN-151758.

### Genetic information

Before sequencing, the estimated genome size, based on ancestral taxa, was 724 Mb, while the estimation based on reads kmer profiling was 479 Mb. Indirect inference of ploidy and haploid number indicate a diploid genome with a haploid number of 17 chromosomes (2n=34), however kmer profiling provides evidence that the genome is tetraploid. Estimates of genome size and karyotype for this species were retrieved from Genomes on a Tree ( Challis *et al*., 2023).

### DNA/RNA processing

DNA was extracted from the anterior body using the Blood & Cell Culture DNA Mini Kit (QIAGEN)Kit (Qiagen) following the manufacturer’s instructions. DNA quantification was performed using a Qubit dsDNA BR Assay Kit (Thermo Fisher Scientific), and DNA integrity was assessed using a Femtopulse system (Genomic DNA 165 Kb Kit, Agilent). DNA was stored at 4ºC until use.

RNA was extracted using an RNeasy Mini Kit (Qiagen) according to the manufacturer’s instructions. RNA was extracted from two different specimen body parts: anterior body and posterior body. RNA quantification was performed using the Qubit RNA BR Kit and RNA integrity was assessed using a Bioanalyzer 2100 system (RNA 6000 Pico Kit,, Agilent). RNA was pooled in a 1:1 ratio before library preparation and stored at -80ºC until use.

### Library preparation and sequencing

A long-read whole genome library was prepared using the SQK-LSK114 kit and sequenced across two R10.4.1 Flow Cells on a PromethION P24 A series instrument (Oxford Nanopore Technologies). For short-read whole genome sequencing (WGS), a library was prepared using the KAPA Hyper Prep Kit (Roche). A Hi-C library preparation, using the head, was conducted with the Dovetail Omni-C Kit (Cantata Bio) and further processed with the KAPA Hyper Prep Kit for Illumina sequencing (Roche). The RNA library, generated from the pooled samples was prepared with the KAPA mRNA Hyper Prep Kit (Roche). All the short-read libraries were sequenced on the Illumina NovaSeq 6000 instrument (2x150bp). In total, 124x Oxford Nanopore, 100x Illumina WGS shotgun, and 160x HiC data were sequenced to generate the assembly.

### Genome assembly methods

**Figure 2.**
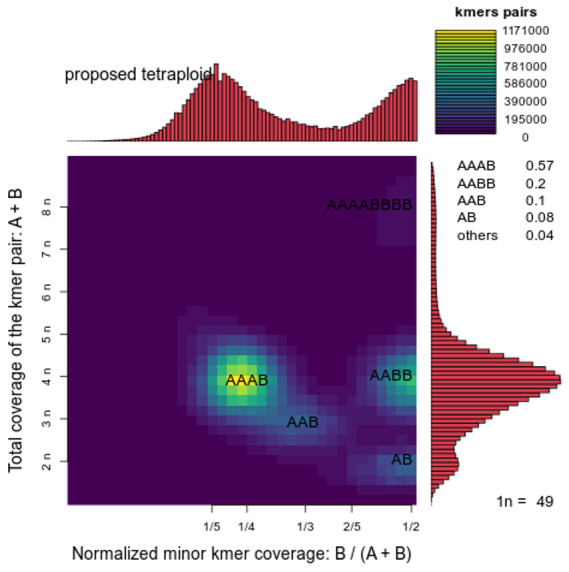
Smudgeplot ploidy estimation. Kmer-based estimation of sample ploidy as calculated by Smudgeplot. The histograms on the x- and y-axes show the normalised ratio of pair-wise kmers and total pair-wise kmer counts, respectively. “Smudges” show increased co-localisation of the two counts, and are used to estimate ploidy.

The genome was assembled using the CNAG CLAWS pipeline v2.2.0 ( Gomez-Garrido, 2024). Briefly, reads were preprocessed for quality and length using Trim Galore v0.6.7 and Filtlong v0.2.1 with parameters --min_length 1000 --min_mean_q 80 -t 61000000000, resulting in a read of N50=19.5 kb and median quality=Q20.6. K-mer analysis with Smudgeplot v0.2.5 ( Ranallo-Benavidez *et al*., 2020) proposed tetraploidy ( Figure 2), thus initial contigs were assembled using HiFiasm v0.24.0 with parameters –ont –n-hap 4 and the primary assembly was chosen for further processing. This was followed by removal of retained haplotigs using purge-dups v1.2.6 and scaffolding with YaHS v1.2a. Finally, assembled scaffolds were curated via manual inspection using Pretext v0.2.5 with the Rapid Curation Toolkit ( https://gitlab.com/wtsi-grit/rapid-curation) to remove any false joins and incorporate any sequences not automatically scaffolded into their respective locations in the chromosomal pseudomolecules (or super-scaffolds). In total 51 haplotigs were removed by purge_dups, while manual curation removed two additional haplotigs from scaffolds and two from the unplaced sequences. Summary analysis of the released assembly was performed using the ERGA-BGE Genome Report ASM Galaxy workflow ( De Panis, 2024a), incorporating tools such as BUSCO v5.5 and Merqury v1.3.

### Genome annotation methods

A gene set was generated using the Ensembl Gene Annotation system ( Aken *et al*., 2016), primarily by aligning publicly available short-read RNA-seq data from BioSamples SAMEA117792403 and SAMEA3303732 to the genome. Gaps in the annotation were filled via protein-to-genome alignments of a select set of clade-specific proteins from UniProt ( The UniProt Consortium, 2019), which had experimental evidence at the protein or transcript level. At each locus, data were aggregated and consolidated, prioritising models derived from RNA-seq data, resulting in a final set of gene models and associated non-redundant transcript sets. To distinguish true isoforms from fragments, the likelihood of each open reading frame (ORF) was evaluated against known metazoan proteins. Low-quality transcript models, such as those showing evidence of fragmented ORFs, were removed. In cases where RNA-seq data were fragmented or absent, homology data were prioritised, favouring longer transcripts with strong intron support from short-read data. The resulting gene models were classified into two categories: protein-coding, and long non-coding. Models that did not overlap protein-coding genes, and were constructed from transcriptomic data were considered potential lncRNAs. Potential lncRNAs were further filtered to remove single-exon loci due to their unreliability. Putative miRNAs were predicted by performing a BLAST search of miRBase ( Kozomara *et al*., 2019) against the genome, followed by RNAfold analysis ( Gruber *et al*., 2008). Other small non-coding loci were identified by scanning the genome with Rfam ( Kalvari *et al*., 2018) and passing the results through Infernal ( Nawrocki & Eddy, 2013). Summary analysis of the released annotation was performed using the ERGA-BGE Genome Report ANNOT Galaxy workflow ( De Panis, 2024b), incorporating tools such as AGAT v1.2, OMArk v0.3, and others (see reference for the full list of tools).

## Results

### Genome assembly

**Figure 3.**
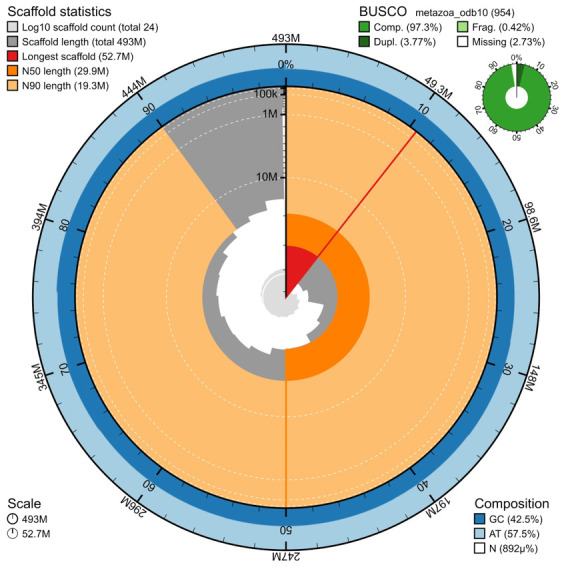
Snail plot summary of assembly statistics. The main plot is divided into 1,000 size-ordered bins around the circumference, with each bin representing 0.1% of the 493,041,447 bp assembly. The distribution of sequence lengths is shown in dark grey, with the plot radius scaled to the longest sequence present in the assembly (52,692,838 bp, shown in red). Orange and pale-orange arcs show the scaffold N50 and N90 sequence lengths (29,876,410 and 19,297,223 bp), respectively. The pale grey spiral shows the cumulative sequence count on a log-scale, with white scale lines showing successive orders of magnitude. The blue and pale-blue area around the outside of the plot shows the distribution of GC, AT, and N percentages in the same bins as the inner plot. A summary of complete, fragmented, duplicated, and missing BUSCO genes found in the assembled genome from the metazoa database (odb10) is shown on the top right.

**Figure 4.**
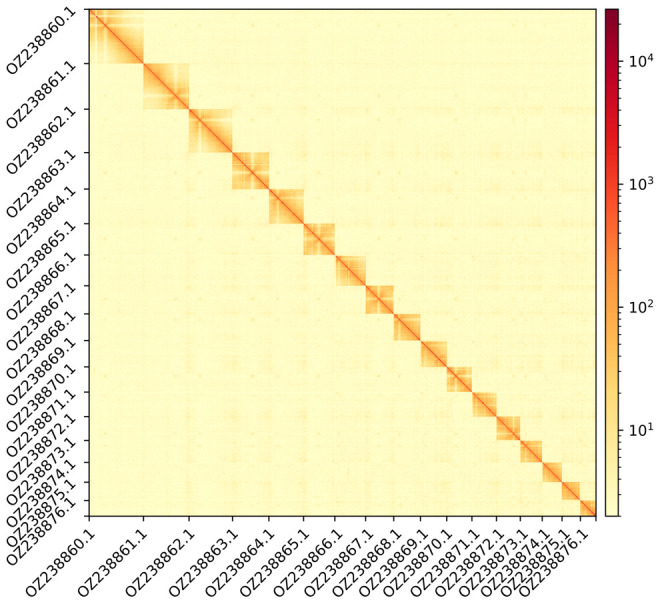
Hi-C contact map showing spatial interactions between regions of the genome. The diagonal corresponds to intra-chromosomal contacts, depicting chromosome boundaries. The frequency of contacts is shown on a logarithmic heatmap scale. Hi-C matrix bins were merged into a 125 kb bin size for plotting.

The genome assembly has a total length of 493,041,447 bp in 24 scaffolds ( Figure 3 and Figure 4), with a GC content of 42.48%. It features a contig N50 of 15,094,000 bp (L50=10) and a scaffold N50 of 29,876,410 bp (L50=7). There are 22 gaps, totaling 4.4 kb in cumulative size. The single-copy gene content analysis using the metazoa_odb10 database with BUSCO resulted in 97.3% completeness (93.5% single and 3.8% duplicated). 56.93% of reads k-mers were present in the assembly and the assembly has a base accuracy Quality Value (QV) of 53.9 as calculated by Merqury.

### Genome annotation

The genome annotation consists of 23,944 protein-coding genes with an associated 40,974 transcripts, in addition to 1,237 non-coding RNA genes of various types ( Table 1). Using the longest isoform per transcript, the single-copy gene content analysis using the metazoa_odb10 database with BUSCO resulted in 95.6% completeness. Using the OMAmer Lophotrochozoa database for OMArk resulted in 95.78% completeness and 61.74% consistency ( Table 2).

**Table 1.** Statistics from assembled gene models.

	No. genes	No. transcripts	Mean * gene length (bp)	No. single-exon genes	Mean * exons per transcript
**Protein-coding**	23,944	40,974	11,551	642	8.7
**lncRNA**	3,032	3,482	4,980	2	2.4
**snRNA**	64	64	156	64	1.0
**snoRNA**	46	46	115	46	1.0
**rRNA**	25	25	119	25	1.0
**tRNA**	224	224	76	224	1.0
**scRNA**	2	2	130	2	1.0
**Other non-coding**	876	876	74-87	876	1.0

*Combined categories show the range of the mean values

**Table 2.** Annotation completeness and consistency scores calculated by BUSCO run in protein mode (metazoa_odb10) and OMArk (Lophotrochozoa).

	Complete	Singular	Duplicated	Fragmented	Missing
**BUSCO**	912 (95.6%)	874 (91.6%)	38 (4.0%)	19 (2.0%)	23 (2.4%)
**OMArk**	2,062 (95.7%)	1,706 (79.2%)	356 (16.5%)	-	91 (4.3%)
	Consistent	Inconsistent	Contaminants	Unknown	
**OMArk**	14,784 (61.7%)	3,066 (12.8%)	0	6,094 (25.5%)	

## Data Availability

*Ailoscolex lacteospumosus* and the related genomic study were assigned to Tree of Life ID (ToLID) 'whAilLact1' and all sample, sequence, and assembly information are available under the umbrella BioProject PRJEB77800 ( Aguilera *et al*., 2024). The sample information is available at the following BioSample accessions: SAMEA115084268, SAMEA115084269 and SAMEA117792403. The genome assembly is accessible from ENA under accession number GCA_965183755.1 and the annotated genome is available through the Ensembl page ( https://beta.ensembl.org/) and ftp site: https://ftp.ebi.ac.uk/pub/ensemblorganisms/Ailoscolex_lacteospumosus/GCA_965183755.1/. Sequencing data produced as part of this project are available from ENA at the following accessions: ERX13167076, ERX14095442, ERX14095443 and ERX12752274. The genome annotation is available from Ensembl under accession number GCA_965183755.1 ( Lazar *et al*., 2025). All data are published under CC0 licence. Documentation related to the genome assembly and curation can be found in the ERGA Assembly Report (EAR) document available at https://github.com/ERGA-consortium/EARs/tree/main/Assembly_Reports/Ailoscolex_lacteospumosus/whAilLact1. Further details and data about the project are hosted on the ERGA portal at https://portal.erga-biodiversity.eu/data_portal/1046305.

## References

[ref-1] Aguilera L Gut M Ferreira FC : *Ailoscolex lacteospumosus* genome sequencing and assembly. European Nucleotide Archive.Project: PRJEB77800 [Dataset],2024.

[ref-2] Aken BL Ayling S Barrell D : The Ensembl gene annotation system. *Database (Oxford).* 2016;2016: baw093. 10.1093/database/baw093 27337980 PMC4919035

[ref-3] Bouché MB : *Ailoscolex lacteospumosus*, n. gen., n. sp., un ver de terre aux caractères morphologiques et biologiques remarquables (Oligochaeta, Ailoscolecidae, nov. fam.). *Rev Écol Biol Sol.* 1969;6(4):525–531. Reference Source

[ref-4] Challis R Kumar S Sotero-Caio C : Genomes on a Tree (GoaT): a versatile, scalable search engine for genomic and sequencing project metadata across the eukaryotic Tree of Life [version 1; peer review: 2 approved]. *Wellcome Open Res.* 2023;8:24. 10.12688/wellcomeopenres.18658.1 36864925 PMC9971660

[ref-6] De Panis D : ERGA-BGE genome report ASM analyses (one-asm WGS Illumina PE + HiC). WorkflowHub , 2024a. 10.48546/WORKFLOWHUB.WORKFLOW.1103.2

[ref-7] De Panis D : ERGA-BGE genome report ANNOT analyses. WorkflowHub, 2024b. 10.48546/WORKFLOWHUB.WORKFLOW.1096.1

[ref-8] Díaz Cosín DJ Ruiz MP Ramajo M : Is the aestivation of the earthworm *Hormogaster elisae* a paradiapause? *Invertebr Biol.* 2006;125(3):250–255. 10.1111/j.1744-7410.2006.00057.x

[ref-9] Gomez-Garrido J : CLAWS (CNAG's long-read assembly workflow in Snakemake). WorkflowHub, 2024. 10.48546/WORKFLOWHUB.WORKFLOW.567.2

[ref-10] Gruber AR Lorenz R Bernhart SH : The Vienna RNA websuite. *Nucleic Acids Res.* 2008;36(suppl_2):W70–W74. 10.1093/nar/gkn188 18424795 PMC2447809

[ref-11] Kalvari I Nawrocki EP Argasinska J : Non-coding RNA analysis using the Rfam database. *Curr Protoc Bioinformatics.* 2018;62(1): e51. 10.1002/cpbi.51 29927072 PMC6754622

[ref-12] Kozomara A Birgaoanu M Griffiths-Jones S : miRBase: from microRNA sequences to function. *Nucleic Acids Res.* 2019;47(D1):D155–D162. 10.1093/nar/gky1141 30423142 PMC6323917

[ref-13] Lavelle P Decaëns T Aubert M : Soil invertebrates and ecosystem services. *Eur J Soil Biol.* 2006;42(Supplement 1):S3–S15. 10.1016/j.ejsobi.2006.10.002

[ref-14] Lazar A Haggerty L Martin F : *Ailoscolex lacteospumosus* genome annotation. Ensembl.GCA_ 965183755.1 [Dataset],2025.

[ref-5] Marchán DF Fernández R de Sosa I : Integrative systematic revision of a Mediterranean earthworm family: Hormogastridae (Annelida, Oligochaeta). *Invertebr Syst.* 2018a;32(3):652–671. 10.1071/IS17048

[ref-15] Marchán DF Fernández R Sánchez N : Insights into the diversity of Hormogastridae (Annelida, Oligochaeta) with descriptions of six new species. *Zootaxa.* 2018b;4496(1):65–95. 10.11646/zootaxa.4496.1.6 30313686

[ref-16] Mazzoni CJ Ciofi C Waterhouse RM : Biodiversity: an atlas of European reference genomes. *Nature.* 2023;619(7969):252. 10.1038/d41586-023-02229-w 37433931

[ref-17] Nawrocki EP Eddy SR : Infernal 1.1: 100-fold faster RNA homology searches. *Bioinformatics.* 2013;29(22):2933–2935. 10.1093/bioinformatics/btt509 24008419 PMC3810854

[ref-18] Novo M Fernández R Andrade SCS : Phylogenomic analyses of a Mediterranean earthworm family (Annelida: Hormogastridae). *Mol Phylogenet Evol.* 2016;94(Pt B):473–478. 10.1016/j.ympev.2015.10.026 26522608

[ref-19] Ranallo-Benavidez TR Jaron KS Schatz MC : GenomeScope 2.0 and Smudgeplot for reference-free profiling of polyploid genomes. *Nat Commun.* 2020;11(1): 1432. 10.1038/s41467-020-14998-3 32188846 PMC7080791

[ref-20] The UniProt Consortium: UniProt: a worldwide hub of protein knowledge. *Nucleic Acids Res.* 2019;47(D1):D506–D515. 10.1093/nar/gky1049 30395287 PMC6323992

