# Supplementary material for: ERGA-BGE Genome of Ailoscolex lacteospumosus Bouché, 1969 – the enigmatic milky worm endemic to the Pyrenees
Source: Open Res Eur. 2026 Jan 26;6:26. [Version 1] doi: 10.12688/openreseurope.22136.1 (PMC12917360; doi:10.12688/openreseurope.22136.1)
Supplement: Peer review history

# Reviewer report: Reviewer response for version 1

**DOI:** 10.21956/openreseurope.23961.r68757

**Published:** 2026-02-18

**Authors:** Jennifer McIntyre

**Affiliations:** 1 University of Glasgow, Scotland, UK

A nice paper detailing the collecting, identification and processing of an unusual earthworm, Ailoscolex lacteospumosus, local to a region in southern France, of both evolutionary, agricultural and conservation interest. Using both ONT and Illumina data (WGS and HiC) the authors have curated a chromosomal genome assembly in 17 scaffolds. They have made an excellent set of genomic and transcriptomic resources available to download and explore online in user-friendly ways.

 "Indirect inference of ploidy and haploid number indicate a diploid genome with a haploid number of 17 chromosomes (2n=34), however kmer profiling provides evidence that the genome is tetraploid." It's not clear how the 'indirect inference' was performed. Please can you explain? Was this from GoaT? 

 Please make it clear how many worms were used to make this genome - was gDNA from all 12 worms pooled for sequencing (and for RNA)? Note that smudgeplot expects individuals (https://github.com/KamilSJaron/smudgeplot/wiki/FAQ), and this can affect interpretation; if pools  were used, please can you check whether the suggested tetraploidy is likely to be correct? 

 "primarily by aligning publicly available short-read RNA-seq data from BioSamples SAMEA117792403 and SAMEA3303732 to the genome." makes it sound like you did not use your own RNA-Seq data, and that the data was from different individuals. Perhaps reword to make it clear that one set was generated in this study and the other was publicly available (SAMEA3303732).

 What software did you use to annotate the genome? 

 On ENA, why do you have sequences from another set for two of the chromosomes (https://www.ebi.ac.uk/ena/browser/view/GCA_965183755.1?show=chromosomes)? i.e. OZ238860.1 1 sequences from set CBCYLV01?

Are sufficient details of methods and materials provided to allow replication by others?

Partly

Is the rationale for creating the dataset(s) clearly described?

Yes

Are the datasets clearly presented in a useable and accessible format?

Yes

Are the protocols appropriate and is the work technically sound?

Partly

Reviewer Expertise:

Genomics, Parasitology, Anthelmintic Resistance, Manual genome assembly (HiC)

I confirm that I have read this submission and believe that I have an appropriate level of expertise to confirm that it is of an acceptable scientific standard, however I have significant reservations, as outlined above.

# Reviewer report: Reviewer response for version 1

**DOI:** 10.21956/openreseurope.23961.r69050

**Published:** 2026-02-14

**Authors:** Huifeng Zhao, Yiming Zhang

**Affiliations:** 1 Langfang Normal University, Langfang, China; 2 Hebei Key Laboratory of Animal Diversity, College of Life Science, Langfang Normal University, Langfang, Hebei, China

I would like to thank the authors for generating and sharing an important genomic resource for an understudied and evolutionarily interesting earthworm species. The availability of a chromosome-level genome for Ailoscolex lacteospumosus represents a valuable contribution to annelid genomics and will be of clear interest to the community.

 My main comments relate to the interpretation of genome ploidy, which is a particular point of interest in this study.

 First, it would be important to clarify whether the long-read sequencing data used for genome assembly and the data used for k-mer–based genome size and ploidy inference were derived from a single individual. As k-mer profiles are highly sensitive to sample composition, mixing individuals or tissues with different levels of heterozygosity or mosaicism could substantially affect ploidy inference and complicate interpretation.

 Second, while k-mer and smudgeplot analyses provide useful indications, genome ploidy inference in earthworms can be complicated by the overall complexity of their genomes. In this context, a brief discussion of the limitations of k-mer–based inference would be valuable. It would also be helpful if the authors could include the k-mer frequency plot used for genome size and ploidy estimation as supplementary material.

 Finally, for completeness and to facilitate comparison with other genome resources, it would be helpful if the authors could report the average proportion of ambiguous bases (Ns) within scaffolds in the final assembly.

 Overall, I believe that addressing these points would further strengthen the clarity and interpretability of this valuable genome report.

Are sufficient details of methods and materials provided to allow replication by others?

Partly

Is the rationale for creating the dataset(s) clearly described?

Yes

Are the datasets clearly presented in a useable and accessible format?

Yes

Are the protocols appropriate and is the work technically sound?

Yes

Reviewer Expertise:

Genomes

We confirm that we have read this submission and believe that we have an appropriate level of expertise to confirm that it is of an acceptable scientific standard, however we have significant reservations, as outlined above.

# Reviewer report: Reviewer response for version 1

**DOI:** 10.21956/openreseurope.23961.r69051

**Published:** 2026-02-10

**Authors:** Arun Arumugaperumal

**Affiliations:** 1 Rajalakshmi Engineering College, Chennai, Tamil Nadu, India

The authors have reported the genome sequence of *Ailoscolex lacteospumosus*, a type of earthworm endemic to the Pyrenees mountain range in Europe.

 The authors have used a combination of Oxford nanopore technology and illumina technology along with Hi-C data to obtain a high quality genome sequence. The assembly and analysis protocols followed are of high standards. The assembly reported here is of size 493 Mb.

 The authors have reported that there are 17 chromosome molecules and the species is tetraploid. The assembly had initially 46 contigs which were scaffolded into 24 scaffolds.

 A total of 23,944 protein coding genes and 1,237 non-coding genes were identified. Only with a high quality genome can reliable protein-coding genes can be obtained. BUSCO analysis with respect to metazoa_odb10 dataset shows that the genome is 97.3% complete. The links provided in the genome note are all active during the time of review. The genome sequence information will be useful for future evolutionary studies. The data note can be indexed.

Are sufficient details of methods and materials provided to allow replication by others?

Yes

Is the rationale for creating the dataset(s) clearly described?

Yes

Are the datasets clearly presented in a useable and accessible format?

Yes

Are the protocols appropriate and is the work technically sound?

Yes

Reviewer Expertise:

Bioinformatics; Genomics

I confirm that I have read this submission and believe that I have an appropriate level of expertise to confirm that it is of an acceptable scientific standard.
